# Antioxidant Synergy of Mitochondrial Phospholipase PNPLA8/iPLA2γ with Fatty Acid–Conducting SLC25 Gene Family Transporters

**DOI:** 10.3390/antiox10050678

**Published:** 2021-04-26

**Authors:** Martin Jabůrek, Pavla Průchová, Blanka Holendová, Alexander Galkin, Petr Ježek

**Affiliations:** 1 Department of Mitochondrial Physiology, Institute of Physiology of the Czech Academy of Sciences, Vídeňská 1084, 14220 Prague, Czech Republic; pavla.pruchova@fgu.cas.cz (P.P.); blanka.holendova@fgu.cas.cz (B.H.); jezek@biomed.cas.cz (P.J.); 2 Department of Pediatrics, Division of Neonatology, Columbia University William Black Building, New York, NY 10032, USA; ag4003@cumc.columbia.edu

**Keywords:** antioxidant synergy, mitochondrial phospholipase A2γ, mitochondrial uncoupling proteins, adenine nucleotide translocase, mitochondrial carriers, SLC25 gene family, lipid peroxidation, cytoprotection

## Abstract

Patatin-like phospholipase domain-containing protein PNPLA8, also termed Ca^2+^-independent phospholipase A2γ (iPLA2γ), is addressed to the mitochondrial matrix (or peroxisomes), where it may manifest its unique activity to cleave phospholipid side-chains from both sn-1 and sn-2 positions, consequently releasing either saturated or unsaturated fatty acids (FAs), including oxidized FAs. Moreover, iPLA2γ is directly stimulated by H_2_O_2_ and, hence, is activated by redox signaling or oxidative stress. This redox activation permits the antioxidant synergy with mitochondrial uncoupling proteins (UCPs) or other SLC25 mitochondrial carrier family members by FA-mediated protonophoretic activity, termed mild uncoupling, that leads to diminishing of mitochondrial superoxide formation. This mechanism allows for the maintenance of the steady-state redox status of the cell. Besides the antioxidant role, we review the relations of iPLA2γ to lipid peroxidation since iPLA2γ is alternatively activated by cardiolipin hydroperoxides and hypothetically by structural alterations of lipid bilayer due to lipid peroxidation. Other iPLA2γ roles include the remodeling of mitochondrial (or peroxisomal) membranes and the generation of specific lipid second messengers. Thus, for example, during FA β-oxidation in pancreatic β-cells, H_2_O_2_-activated iPLA2γ supplies the GPR40 metabotropic FA receptor to amplify FA-stimulated insulin secretion. Cytoprotective roles of iPLA2γ in the heart and brain are also discussed.

## 1. Introduction

Cellular redox homeostasis, related redox regulations, and antioxidant mechanisms may rely not only on reactions of a single protein but also on cycling or synergy reactions of two or several proteins. Since, in numerous types of eukaryotic cells and/or mammalian tissues, mitochondria are dominant sources of reactive oxygen species (ROS) and in certain tissues occupy a rather substantial cell volume, nature has developed mechanisms that prevent excessive ROS production. Therefore, multiple antioxidant systems were evolved within mitochondria to interact with (react to) internally produced ROS [1,2,3,4,5,6,7,8,9].

Regulatory mechanisms attenuating redox signaling have also been phylogenetically developed [6]. Redox signaling is substantiated when a mild excessive ROS production occurs during a specific time interval. In principle, pathological development of oxidative stress and its consequences differ from physiological regulatory mechanisms not only by the amplitude (strength) of ROS elevation but may be distinct also by the reaction mechanism or pathways involved. The oxidative stress (alternatively, the creation of a redox signal) can be ensured either by the extra prolonged timing or by the excessively/relatively high ROS formation, respectively, or by the diminished detoxification of given ROS species, i.e., diminished antioxidant action. These actions may act in combinations and/or simultaneously.

The machinery of oxidative phosphorylation (OXPHOS) residing in mitochondria has been developed to contain antioxidant mechanisms inherently. Respiratory chain proton pumping across the inner mitochondrial membrane (IMM) into the intracristal space (ICS) creates the protonmotive force Δp (Δp = ΔΨ_m_ + ΔpH), which is used by the ATP-synthase for rotation of its membrane c-ring and for the synthesis of ATP. A mild dissipation of Δp by a moderate simultaneous H^+^ backflow into the mitochondrial matrix, termed mild uncoupling, consequently leads to a decrease in ROS formation (representing an antioxidant action) due to releasing the previously retarded path of electron transport. Such retardation normally occurs in the proximity of the “toxic” sites of superoxide formation within the respiratory chain complexes [5]. Such a mild uncoupling, however, cannot be effective when mutated mitochondrial mtDNA provides these retardations, i.e., when being caused by mutant subunits encoded by mtDNA.

Mild uncoupling can be regulated by mitochondrial uncoupling proteins (UCPs). Furthermore, it has long been known that certain solute carriers residing in the mitochondrial inner membrane, other than UCPs, are able to provide uniport of negatively charged fatty acid anions [10,11]. Together with UCPs, they belong to the SLC25 gene family of mitochondrial carriers. In fact, the ADP/ATP carrier (or adenine nucleotide translocase, ANT) was the first carrier for which the H^+^ transport by the so-called fatty acid cycling mechanism was predicted by Skulachev in 1991 [10]. The ability of fatty acids (FAs) to induce ANT-dependent H^+^ transport was more recently verified by sophisticated patch-clamp measurements [12], similarly as for UCPs [13]. In vivo or patch-clamp in vitro studies also support the concept of nascent fatty acids and the instant spatiotemporal release of FA molecules within the IMM bilayer after cleavage from phospholipids, such as cardiolipin (or income as being unbound from protein carries, enzymes, etc.) [13].

It has also been recognized that the IMM lipids or an inner phospholipid leaflet of the outer mitochondrial membrane (OMM) are targets of phospholipases A2 [14,15,16]. Unidentified mitochondrial isoforms studied in the past were eventually recognized as phospholipase A2 isoforms PNPLA8/iPLA2γ and probably also PNPLA9/iPLA2β (see below). Nevertheless, why consider phospholipases in relation to the antioxidant action? It is because any phospholipase, which may reside either in the intermembrane or in the matrix side of IMM, such as iPLA2γ, may efficiently cleave mitochondrial phospholipids and release free FAs. As a result, mitochondrial iPLA2γ can supply nascent FAs for UCPs and other SLC25 mitochondrial carrier family members. Subsequently, these synergic protein couples provide mild dissipation of Δp, leading to a decrease of mitochondrial superoxide production and an antioxidant action [17,18,19,20].

In this review, we first explain the molecular mechanism of antioxidant synergy involving iPLA2γ and UCPs and selected cases of the SLC25 family proteins. Then, we point out iPLA2γ relations to specific IMM lipid composition, given by exclusive content of cardiolipin, and mention specific iPLA2γ roles and activation by lipid peroxidation. Finally, we discuss already recognized cases of participation of iPLA2γ in antioxidant mechanism and/or cytoprotection in vivo in selected tissues and organs.

## 2. Phospholipase A2 Group VI, Patatin-Like Phospholipase Domain-Containing Proteins (PNPLAs)

### 2.1. Classification of Phospholipases A2

In the mammalian genome, more than 30 different mammalian phospholipase A2 (PLA2) enzymes were identified and subdivided into several classes. Overview of selected phospholipases with the focus on group VI intracellular calcium-independent phospholipases A2 is shown in Table 1, below.

**Figure 1:**
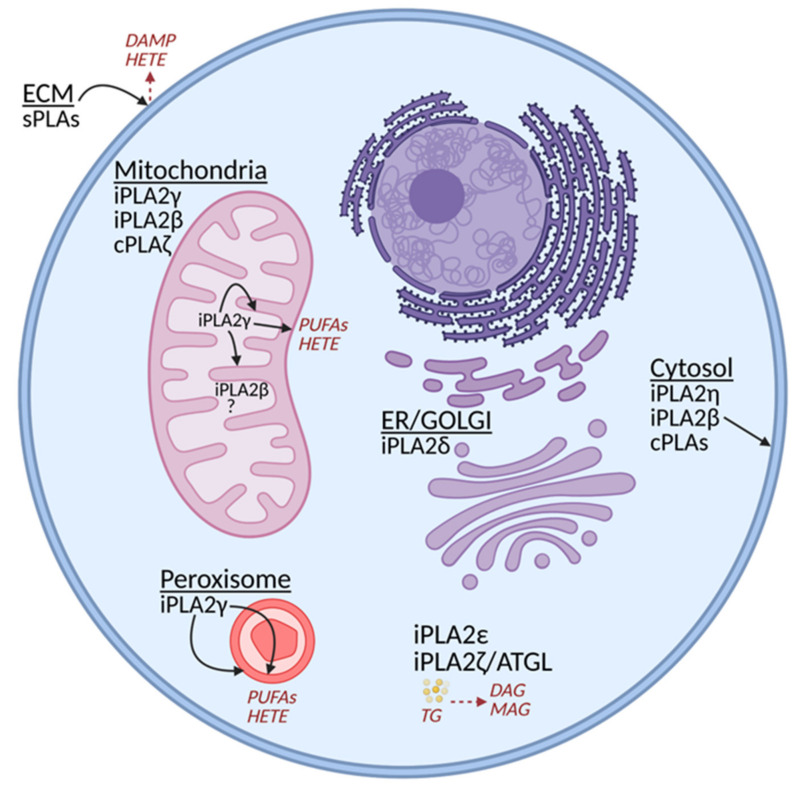
Scheme of the distribution of phospholipase A2 family members within the cell. Selected cleavage products are also illustrated. DAG, diacylglycerol; DAMP, Danger-Associated Molecular Pattern; ECM, extracellular matrix; HETE, 12-hydroxyeicosatetraenoic acid; MAG, monoacylglycerol; PUFA, polyunsaturated fatty acid; TG, triglycerides. (The illustration was created with the aid of BioRender.com.)

Groups I–III, V, X, and XII are represented by the secretory PLA2s (sPLA2s), which are secreted into the extracellular milieu. Consequently, they frequently help with the remodeling of the extracellular matrix (ECM), and their reactions release specific lipid mediators and participate in lipid metabolism [21]. Of course, neither of sPLA2s can be regarded as a mitochondrial PLA2. Group IV then contains Ca^2+^-dependent and arachidonyl-specific PLA2s residing in the cytosol. In principle, they could also affect the outer phospholipid leaflet of OMM (Figure 1).

Group VI includes patatin-like phospholipase domain-containing lipases, PNPLAs [14], which are Ca^2+^-independent iPLA2s containing lipase (GXSXG) and nucleotide-binding (GXGXXG) consensus sequences. Generally, PLA2s release FAs by cleaving the sn-2 ester bond of membrane phospholipids leaving lysophospholipids in the membrane. The Group IV phospholipases also cleave phospholipid acyl chains from both sn-1 and sn-2 positions of phospholipids. Different isoforms of Group VI iPLA2s possess a diverse spectrum of activities in addition to the phospholipase A2 activity, which makes it possible for some of them to act as lysophospholipase, transacylase, hydrolase, or thioesterase [14]. They are ubiquitously expressed in all tissues and cell types while participating in numerous processes, such as phospholipid remodeling, fat catabolism, cell differentiation and proliferation, signal transduction, and cell death. The phospholipase and lysophospholipase PNPLA6/iPLA2δ is predominantly expressed in neurons and located in the endoplasmic reticulum and Golgi apparatus [22]. The hepatocyte PNPLA3/iPLA2ε, rather adipose tissue-specific PNPLA2/iPLA2ζ/ATGL, and ubiquitous PNPLA4/iPLA2η isoforms also manifest triglyceride lipase and acylglycerol transacylase activities [23,24,25]. In this review, we focus on only those phospholipases A2 functioning mainly inside mitochondria, typically in parallel with some other intracellular locations. These isoforms also participate in maintaining mitochondrial integrity.

Concerning the intracellular localization, we must note that any of the cytosolic (cPLA2s) enzymes of Groups IV and VI may be effective onto the cytosolic surface (outer leaflet) of OMM without having the mitochondria-addressing sequence, i.e., without being mitochondria-specific. In contrast, the only Group VI PLA2 enzyme known to contain the N-terminal mitochondrial localization sequence (besides the peroxisomal one) is PNPLA8, also termed iPLA2γ [14,26]. Nevertheless, another widely studied PLA2 isoform PNPLA9/iPLA2β was reported to act in mitochondria [27] and to participate in mitochondrial membrane repair following lipid peroxidation [28]. Recently, the Group IV cPLA2ζ has also been implicated to act on heart mitochondria [29].

### 2.2. Phospholipase PNPLA9/ iPLA2β

The iPLA2β isoform manifests PLA2/PLA1 and lysophospholipase activities, in addition to its transacylase activity [30]. In addition, thioesterase activity was reported for this enzyme [31]. Moreover, 85 kDa of rat iPLA2β protein contains 751 amino acids [32]. The GTSGT serine lipase consensus sequence is positioned after the eight copies of N-terminal ankyrin repeats. The structure of iPLA2β was resolved [33]. In the revealed iPLA2β crystal structure, the open conformation occurred for the active site when the dimeric enzyme was not interacting with the membrane. Open conformation describes that both active sites provide sufficient space for phospholipids to reach the active centers. Nevertheless, this does not allow interfacial activation by simple membrane contacts. The ankyrin domains are oriented outward of the catalytic site/membrane interface. The dimer contains a single calmodulin-binding site, the occupation of which promotes inhibition by narrowing the catalytic site. Since the catalytic centers are ultimately proximal to the interface of the two monomers, the dimerization state affects the activity. Hypothetically, the ankyrin repeats interact with membrane proteins, providing binding to specific membrane loci. Molecular dynamics simulations were conducted for a predicted structure of the common PNPLA monomeric enzyme, demonstrating that a driving force for the attachment of the enzyme to the membrane is favored by electrostatic and non-polar interactions [34]. The iPLA2β enzyme was considered to be located in mitochondria by several authors. The mitochondrial targeting sequence of iPLA2β has been suggested but was not explicitly identified and has remained speculative [27]. Therefore, it is not conclusive whether iPLA2β is imported at all into the mitochondrial matrix [35]. 

### 2.3. Phospholipase PNPLA8/iPLA2γ

**Figure 2:**
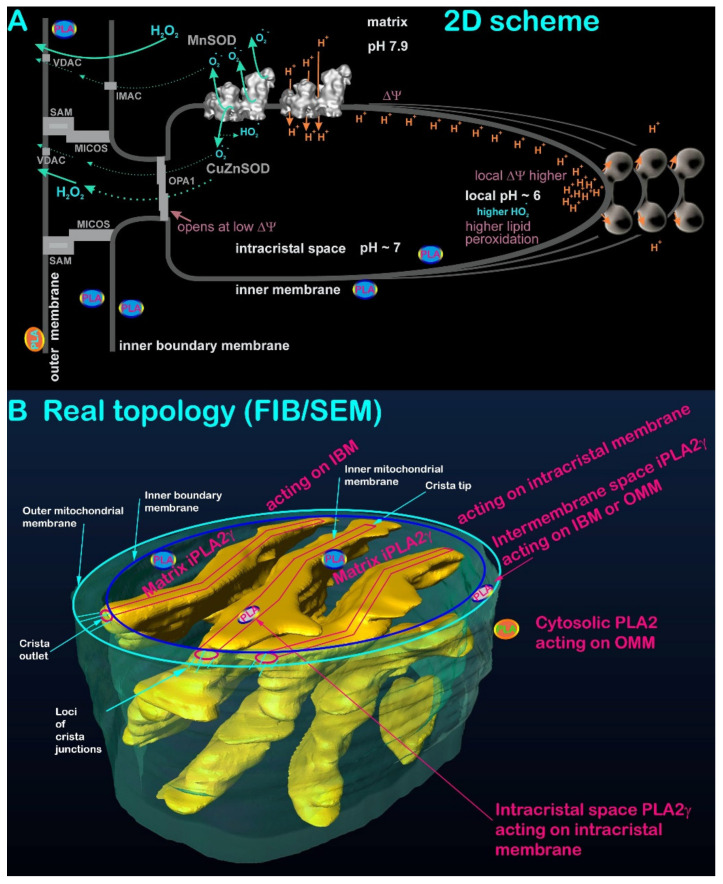
(**A**) Traditional 2D scheme of mitochondrial cristae (modified from Reference [41]) and (**B**) actual 3D topology of cristae within a segment of mitochondrial network tubule (OMM, green) with all possible localizations of mitochondrial phospholipase. The FIB/SEM (focused ion beam/scanning electron microscopy) 3D image of the mitochondrial tubule segment of the mitochondrial network in HepG-2 cells was acquired as described in Reference [42]. The 3D image shows cristae, including the intracristal space as rendered by yellow surface lamellae, representing intracristal membranes (an invaginated IMM portion) together with coated proteins of ATP-synthase and respiratory chain supercomplexes. Red lines illustrate the probable location of the membrane (IMM, not in scale), red ellipses depict the crista outlets, in reality, surrounded by MICOS–SAM joined supercomplexes (cf. Panel A), which are indicated only by thin blue lines. Note that intracristal or intermembrane space location for iPLA2γ is hypothetical. The latter location must exist if iPLA2γ participates in remodeling of cardiolipin acyl chains (see Section 5).

The mammalian iPLA2γ mRNA contains four translation initiation sites from which potentially 88-, 77-, 74-, and 63-kDa isoforms may arise. Among them, the 63-kDa protein was characterized as peroxisomal [26,36]. Nascent isoforms containing the N-terminal mitochondrial localization sequence are imported into the mitochondrial matrix, where the addressing sequence is cleaved off. Therefore, instantly after the import, iPLA2γ may affect the IMM inner lipid leaflet (Figure 2). It is not known whether other types of protein import mechanism allow locations of iPLA2γ in the intracristal space or the outer intermembrane space, i.e., in the layer between OMM and inner-boundary membrane (IBM; the part of IMM comprising the cylindrical portion parallel to the cylindrical OMM of mitochondrial network tubules) (Figure 2). The iPLA2γ mRNA was found in multiple human parenchymal tissues, including skeletal muscle, heart, placenta, brain, liver, and pancreas [37]; and in lymphocytes [38].

**Figure 3:**
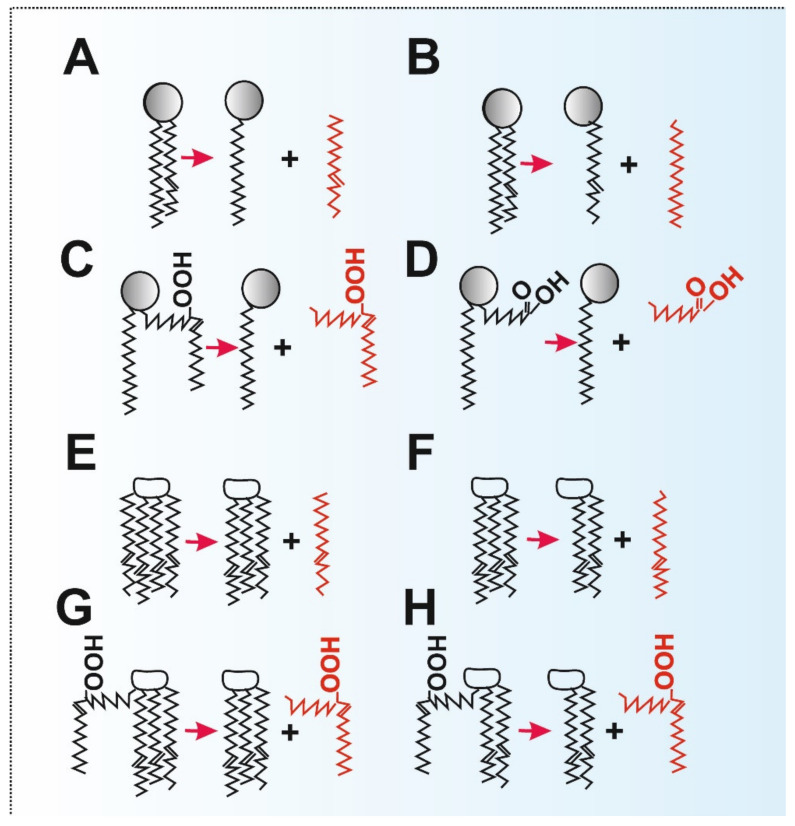
Cleavage modes of iPLA2γ: (**A**) cleavage of unsaturated FA from the sn-2 position (PUFA); (**B**) cleavage of saturated FA from the sn-1 position; (**C**) cleavage of hydroperoxy FA (PUFA after lipid peroxidation) from the sn-2 position; (**D**) cleavage of the shortened alkyl chain (resulting from lipid peroxidation of PUFA and subsequent degradation of the hydroperoxy-alkyl chain); (**E**) cleavage of unsaturated FA (PUFA) from the sn-1 position of cardiolipin; (**F**) cleavage of unsaturated FA (PUFA) from the sn-2 position of monolysocardiolipin; (**G**) cleavage of hydroperoxy FA (PUFA after lipid peroxidation) from the sn-1 position of cardiolipin; (**H**) cleavage of hydroperoxy FA (PUFA after lipid peroxidation) from the sn-2 position of monolysocardiolipin.

**Figure 4:**
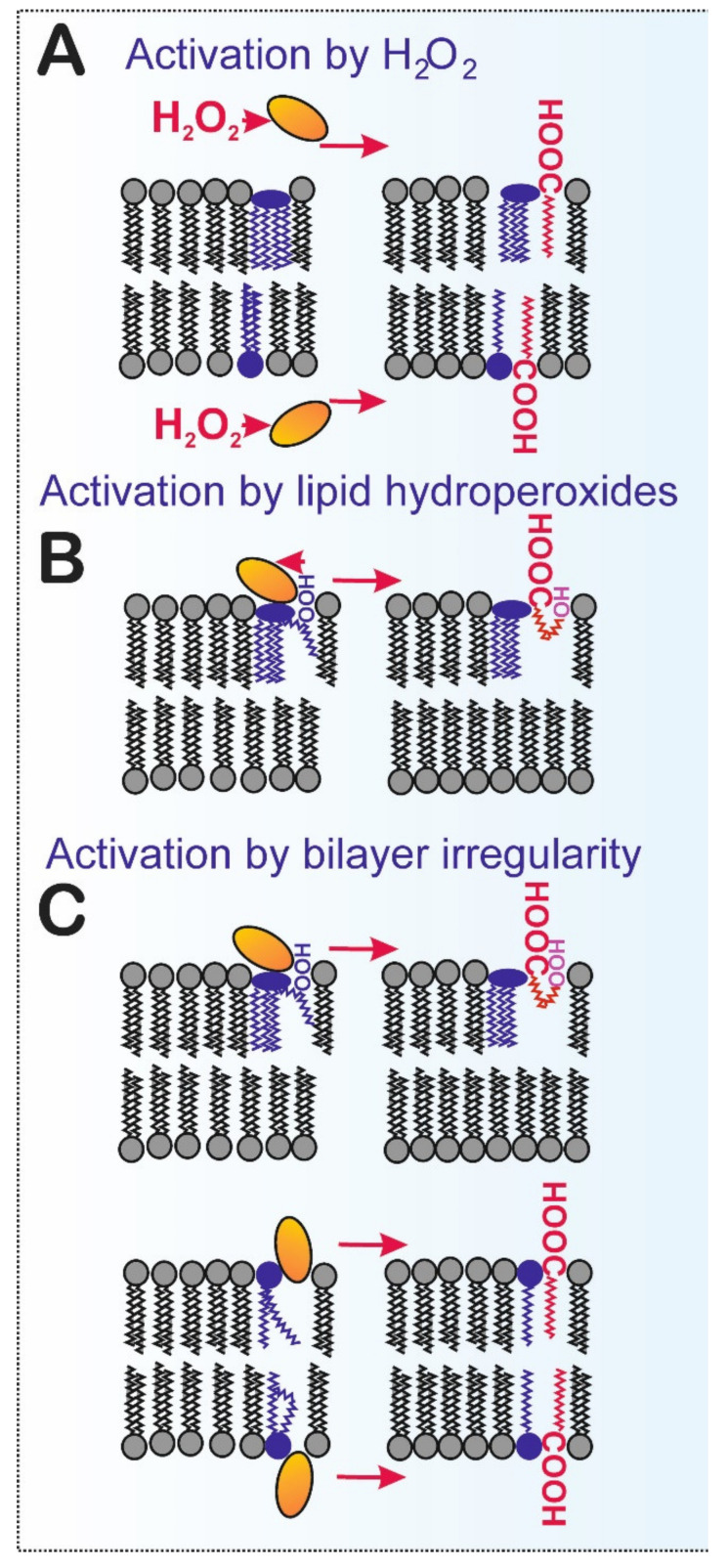
Identified mechanism and hypothetical alternative pathways of iPLA2γ activation. The possible direct redox and indirect redox mechanisms are illustrated. (**A**) Direct activation by H_2_O_2_ was demonstrated by using recombinant iPLA2γ reconstituted in phospholipid vesicles [19] and isolated rodent mitochondria [17,18]; (**B**) activation by cardiolipin hydroperoxides. Lipid hydroperoxides and other lipophilic electrophiles could hypothetically interact with the accessible redox-sensitive site similarly to an H_2_O_2_ molecule, leading to direct oxidation of iPLA2γ thiols [40]. (**C**) Hypothetical activation by structural alteration(s) of the lipid bilayer, caused, for example, by cardiolipin hydroperoxides without direct oxidation of iPLA2γ. These alterations can further be caused by polyunsaturated fatty acids and even hydroxy or peroxy groups existing in phospholipid fatty acyl side-chains and localized at the water/lipid interface.

The iPLA2γ activity is unique in having the ability to cleave phospholipid acyl chains from both sn-1 and sn-2 positions of phospholipids (but not in dilyso-cardiolipin) (Figure 3). Thus iPLA2γ also cleaves off the sn-1 saturated side-chain [35]. An FA cleavage pattern can be indicative of the given PLA2 isoform [19]. For example, purified overexpressed iPLA2γ was found to cleave saturated and monounsaturated FAs at equal amounts from the respective sn-1 and sn-2 positions [35]. The sn-1 cleavage of phospholipids having polyunsaturated FA (PUFA) in the sn-2 side-chain leaves polyunsaturated sn-2-acyl lysophospholipids, which can be processed by 12-lipoxygenase (12-LOX) to products such as 12(s)-hydroxy-eicosatetraenoic (HETE) lysophosphatidylcholine or 12(s)-HETE lysophosphatidyletanolamine [39]. These specific lipid species may subsequently act as lipid signaling mediators important in immune and endothelial cells. Therefore, we can emphasize just another role of iPLA2γ, which is the formation of lipid signaling molecules. Until we observed the direct activation of iPLA2γ by H_2_O_2_ (Figure 4) [17,18,19], the Ca^2+^ independence of the enzyme activity could theoretically be considered as that the enzyme is active when it encounters specific structural alterations in the lipid bilayer. Such alterations can be caused by PUFAs, cardiolipin, and even hydroxy or peroxy groups existing in phospholipid FA side-chains and usually localized at the water/lipid interface. In the latter case, lipid hydroperoxides and other lipophilic electrophiles could hypothetically also interact with the activating site similarly as a H_2_O_2_ molecule (Figure 4) [40].

Unlike for iPLA2β, the structure of iPLA2γ is unknown. The complete transcript leads to 88 kDa protein of 782 amino acids in humans. The iPLA2γ mutants Ser483Ala and Asp627Ala were reported as non-functional, reflecting Ser-Asp dyads as contributing to the catalytic site [43]. (E)-6-(bromomethylene)-3-(1-naphthalenyl)-2H-tetrahydropyran-2-one, also termed R-bromoenol lactone (R-BEL), is a highly selective inhibitor of iPLA2γ, whereas the S-enantiomer (S-BEL) is ineffective, but is highly selective for iPLA2β [37]. The maintenance of lipid homeostasis of mitochondria and peroxisomes is consensually considered as a general function of iPLA2γ [16].

The iPLA2γ plays an essential role in the hydrolysis of oxidized cardiolipin, from which specifically 9-hydroxy-octadecenoic acid was hydrolyzed [44]. Therefore, this could again provide specific lipid mediators in addition to facilitation of clearance of the products of lipid peroxidation. Even without cleavage of oxidized lipids, a general function of iPLA2γ could be remodeling the mitochondrial membranes, as well as the membranes of peroxisomes. Certain studies also support the role of iPLA2γ in specific biosynthetic and regulatory pathways of lipid mediators involving PUFAs, PUFA-lysophospholipids, and monolyso- and dilyso-cardiolipin (see Section 5). Furthermore, iPLA2γ could act as a critical specific regulatory nodule in these particular pathways. For example, in this way, iPLA2γ was suggested to contribute to prostaglandin synthesis in skeletal muscle [45]. Recently, it was shown that high-fat diet activates liver iPLA2γ generating eicosanoids (specifically 12-HETE), leading to mitochondrial dysfunction and hepatic cell death [46].

### 2.4. Redox Activation of Phospholipase iPLA2γ 

**Figure 5:**
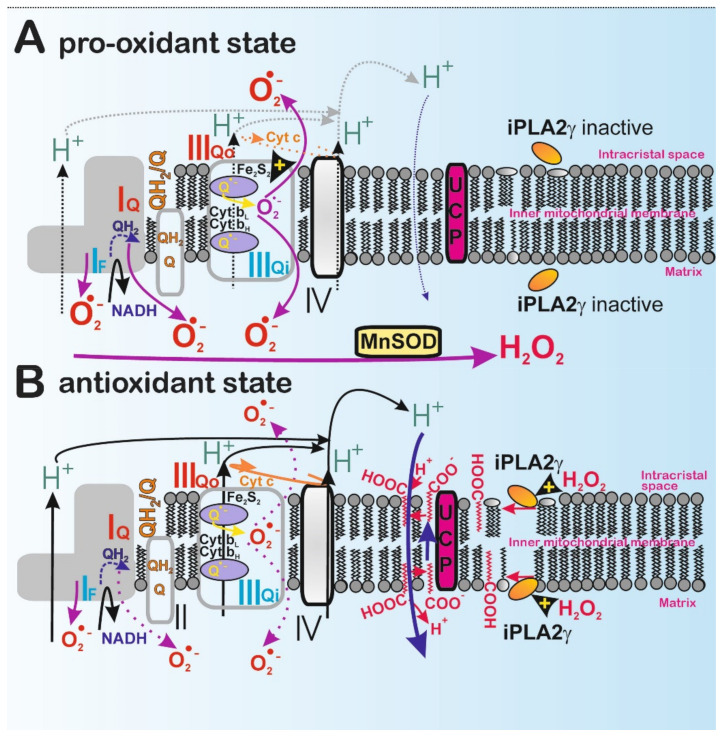
The antioxidant synergy of H_2_O_2_-activated iPLA2γ and UCP2. (**A**) Pro-oxidant state: low respiration, slow H^+^ pumping, and relatively highly coupled state of mitochondria can lead to higher superoxide formation, for example, at sites classified by M. Brand, such as Complex I flavin site I_F_, Complex I ubiquinone (Q) site I_Q_, and Complex III outer ubiquinone site III_Qo_ [67]. In the absence of a mild uncoupling due to the lack of fatty acids, there is no additional acceleration of H^+^ pumping and respiration. (**B**) Antioxidant state: accumulated H_2_O_2_ (transformed from superoxide by MnSOD) activates the mitochondrial phospholipase iPLA2γ, which subsequently cleaves FAs from phospholipids and cardiolipin of the IMM. The nascent FAs bind to UCP2 and are exported as FA anions from the inner (matrix side) to the outer (crista lumen) intracristal membrane phospholipid leaflet [47,48,49]. The subsequent protonation allows FA to cross the lipid bilayer by electroneutral diffusion (flip-flop) back to the matrix, releasing H^+^. Note that protons are not conducted by UCP but by protonated FAs. As a result, a moderate H^+^ short-circuit of the IMM substantiates mild uncoupling, which immediately accelerates electron transport (respiration), leading to a decrease in superoxide formation [18,19].

A significant key feature required for the function and antioxidant action of iPLA2γ is its redox activation. The iPLA2γ was reported to be directly stimulated by H_2_O_2_, and hence it could be activated by redox signaling or oxidative stress [17,18,19]. After redox activation, the antioxidant synergy of UCPs plus iPLA2γ or other SLC25 family members plus iPLA2γ leads to a mild dissipation of Δ*Ψ*_m_. Therefore, mitochondrial superoxide formation diminishes and returns the cell redox state to the level prior to the redox activation (Figure 5). During this process, the concomitantly released unsaturated FAs or PUFAs and saturated FAs interact with mitochondrial uncoupling proteins or certain SLC25 carrier family members, which allow the transport of FA anions across the membrane [5,12,13]. This leads to FA cycling when the return of protonated FAs ensures the H^+^ transport [12,47,48,49,50,51,52,53] back to the mitochondrial matrix (Figure 5). This leads to a mild uncoupling and, consequently, to the attenuation of mitochondrial superoxide formation (reviewed in Reference [5]).

This mechanism can also be part of the suppression of the amplitude of ongoing redox signaling, such as required for cytokine production in the immune cells [5]. Both events thus constitute the reported antioxidant and anti-inflammatory synergy of iPLA2γ plus UCP2 [18,19]. In tissues with less abundant expression of various UCP isoforms (UCP1 to UCP5), the ANT (ADP/ATP carrier) may substitute the UCP role [17]. The synergy of iPLA2γ-ANT should exist in all tissues or cell types, using OXPHOS, since ANT is universally expressed and the most abundant protein of the inner mitochondrial membrane [54]. Evidently, FAs are necessary for H^+^ transport mediated by both ANT and UCPs [5,12,47,48,49,50,51,52,53].

The cytoprotective features of iPLA2γ were suggested for the heart [17] and reported previously in kidney podocytes, preventing glomerular injury [55,56] and pancreatic β-cells [19] and human glioblastoma A172 cells [57]. Activation of iPLA2γ by oxidative stress was reported for the myocardial tissue as concomitant hydrolysis of oxidized cardiolipin [44]. The iPLA2γ was also identified as the membrane potential sensitive phospholipase in liver mitochondria [58]. Given the redox-dependent regulation of iPLA2γ activity and the dependence of mitochondrial ROS production on ΔΨm, this phenomenon calls for further investigation.

## 3. Antioxidant Synergy of iPLA2γ and Mitochondrial Uncoupling Proteins

### 3.1. Nascent FAs—Spatiotemporal FA Release Initiates Mild Uncoupling by UCPs

In the complex cellular environment, fatty acids may arise and reside within the lipid bilayer membranes, such as OMM or IMM, after being released from FA binding proteins or enzymes of FA metabolism, such as from the CD36 FA transporter or long-chain acyl-CoA synthetase (ACSL). Alternatively, FAs are cleaved by mitochondrial phospholipases A2, such as iPLA2γ, from phospholipid/cardiolipin bilayers of IMM and OMM (Figure 1).

As mentioned above, it was concluded from particular observations [13,19] that mild uncoupling activity of UCPs is not initiated by the “bulk” free FAs contained within the IMM, but by “nascent” FAs, that is, FAs cleaved off phospholipids, including cardiolipin of IMM. Patch-clamp studies of the native IMM of brown adipose tissue (BAT) mitochondria indicated currents originating from a high content of UCP1, dependent on a calcium-independent phospholipase initiation [13]. The identity of the phospholipase(s) was not resolved.

In our studies of insulinoma INS-1E cells with silenced iPLA2γ, we did not observe any mild uncoupling resulting from the simple addition of exogenous FA to the cell suspension, which otherwise existed in controls transfected with scrambled siRNAs [19]. Therefore, the latter mechanism was explained in terms of redox activation of iPLA2γ by ROS produced during the β-oxidation of the added exogenous FA and by the inability of “bulk” FA added to cells to directly induce UCP2-mediated uncoupling. It was further verified that the ROS-induced mild uncoupling was absent following UCP2 silencing [19].

### 3.2. Mechanism of Uncoupling Protein-Mediated Suppression of Mitochondrial Superoxide Formation

Mitochondrial uncoupling proteins dissipate Δp and thus uncouple respiratory chain proton pumping from ATP synthesis, driving the proton backflux into the matrix via the rotating c-ring of the ATP-synthase membrane F_O_ moiety. This dissipation of Δp can be extensive, such as in the case of UCP1 of BAT mitochondria, where UCP1 provides a nearly complete “thermogenic” uncoupling towards low Δp values [59]. The term “mild uncoupling” has been introduced as a part of membrane-linked systems preventing superoxide formation when Δp is dissipated only partially [60]. During this process, the value of Δp is still high enough to drive ATP synthesis, while the parallel H^+^ backflow constitutes the uncoupling (H^+^ leak). It should be emphasized that intensity of H^+^ leak across the inner membrane is non-linear, following non-ohmic behavior. As the Δp (usually measured as ΔΨ_m_) decreases, disproportionately large changes in the rate of oxygen consumption become apparent to defend the protonmotive force [61,62]. On the contrary, during ATP synthesis when the H^+^ flux is high, and ΔΨm is relatively low, any increase in the H^+^ transport mediated, for example, by uncoupling proteins, may lead to only a small increase in respiration, but the corresponding decrease in ΔΨm would be relatively high [61,63,64].

After decades of controversy, FAs are now consensually established as the entity required for such mild uncoupling [12,13,65,66]. Hence, independently of the molecular mechanism involved, mild uncoupling is initiated by FAs, most likely endogenously released by mitochondrial phospholipases.

When electron transport by the respiratory chain is locally retarded, superoxide formation is increased [67] (Figure 5A). This situation occurs under so-called coupled respiration, because the capacity of the respiratory chain H^+^ pumping is in excess over the H^+^ backflow via the c-ring of ATP-synthase. Under these conditions, ΔΨ_m_ is high. Therefore, ROS formation was found to be strongly potential-dependent [68,69,70].

Retarded electron transport (respiration) was related to a phenomenon of so-called respiratory control. Originally, an increase of respiration by ADP was defined as the release of respiratory control. A molecular explanation for this phenomenon was presented by the chemiosmotic hypothesis postulating Δp of oxidative phosphorylation as an intermediate that is consumed by the ATP-synthase for the formation of ATP from ADP and phosphate.

In contrast, mild uncoupling by free fatty acids would also decrease Δp and therefore attenuate ROS generation by the respiratory chain (Figure 5B). Once the steady-state is disturbed by an additional H^+^ backflow (H^+^ leak), enabled, for example, by FA-dependent UCP-mediated H^+^ transport, the proton pumping and electron transport are less inhibited by Δp, which accelerates electron flow at the sites, where superoxide can be formed. Faster electron translocation decreases the chances of superoxide formation (Figure 5B). This is the main principle explaining why mild uncoupling leads to the attenuation of mitochondrial superoxide formation. It is interesting to note that proton conductance by free FAs in the absence of uncoupling proteins is seen in planar membranes and is strongly dependent on the FA molar fraction in the membrane-forming solution [71]. This suggests that a rise in FA concentration combined with the alteration in transmembrane potential may also contribute to the FA-mediated proton leak and attenuate ROS generation under specific physiological or pathological conditions [71].

Note, however, this mechanism cannot act when mutations of either Complex I or Complex III proton-pumping machinery exist, which automatically irreversibly retard proton pumping and electron transfer, respectively. It was just nature’s logistics during evolution that the key ND subunits of Complex I proton pumping and Complex III subunits are encoded by mitochondrial DNA. That is why mild uncoupling cannot rescue higher superoxide formation established due to mutations of these mtDNA encoded subunits [72,73,74], and neither can mild uncoupling rescue high superoxide formation in so-called mutator mice (having impaired mtDNA polymerase, i.e., Polγ) [75].

### 3.3. Antioxidant Synergy of iPLA2γ and Uncoupling Proteins

The theoretical principles described above were confirmed, namely for the antioxidant synergy of the mitochondrial uncoupling protein UCP2 and mitochondrial phospholipase iPLA2γ [18,19]. Upon initiated function of both these proteins, indeed, downregulation of mitochondrial superoxide production was observed. Experiments were relying on the discovered ability of iPLA2γ to be redox-activated. The mechanism of redox activation is discussed in detail in Section 4. 

Experimental evidence supports the existence of the antioxidant synergy of iPLA2γ with UCP2, which downregulates oxidative stress in lung and spleen, tissues in which UCP2 expression is most abundant [18]. Thus, typically to activate iPLA2γ, *tert*-butyl hydroperoxide (TBHP) or H_2_O_2_ are added to isolated mitochondria or cultured cells. TBHP and H_2_O_2_ induce nascent FA release from mitochondrial membranes, which is inhibited by R-BEL but not by its stereoisomer S-BEL, indicating the participation of iPLA2γ. TBHP and H_2_O_2_ cause an increase in respiration and a decrease in Δ*Ψ*_m_ in mitochondria from control, but not UCP2-knockout mice. Both changes are again completely inhibited by r-BEL, a selective inhibitor of calcium-independent phospholipases iPLA2γ [17,18]. Alternatively, iPLA2γ is activated by palmitoylcarnitine, which can be explained by the H_2_O_2_ produced in conjunction with the electron-transferring flavoprotein:ubiquinone oxidoreductase (ETFQOR) of FA β-oxidation.

In isolated heart mitochondria, the hydroperoxide-initiated uncoupling is inhibited by carboxyatractyloside and purine di- and tri-phosphates, indicating that the observed mild uncoupling originated from redox-activated iPLA2γ providing nascent FAs for the onset of FA-dependent H^+^ transport mediated by the ANT1 and UCP(s) [17]. In similar experiments with mitochondria isolated from tissues rich in UCP2 [18] and insulinoma INS-1E cells [19], the antioxidant synergy of UCP2 plus iPLA2γ was demonstrated being dependent on the identified redox activation of iPLA2γ. The data support the mechanism of H_2_O_2_-activated iPLA2γ, leading to subsequent cleavage of phospholipids and release of free nascent FAs that are the cycling substrates of UCP2, causing the mild uncoupling. The consequent partial dissipation of Δp has been shown to initiate a direct feedback attenuation of mitochondrial superoxide production [18,19,20].

### 3.4. Mutual Influence of Mitochondrial and Cytosolic Redox State

In tissues where the mitochondrial network accounts for a substantial cell volume, any shift in the redox state of the mitochondrial matrix must be reflected in the cell cytosol or even outside the cell [1]. Therefore, when the decreased rates of H_2_O_2_ and superoxide release from mitochondria to the cytosol are established, this decrease leaves a spare cell antioxidant capacity that had previously been used to detoxify mitochondrial H_2_O_2_ and superoxide. The additional detoxification capacity can then act on other cellular processes, e.g., to slow down lipid peroxidation and protein and DNA oxidation. In this way, the diminished mitochondrial ROS source is spread in the form of antioxidant protection of the cell and allows for the maintenance of the steady-state redox status of the cell.

From what was stated above, we propose an essential physiological role for the iPLA2γ in the overall cellular redox homeostasis. The couple iPLA2γ/UCP allows a feedback downregulation not only of mitochondrial superoxide production, but also of cellular redox status. Any pro-oxidative insult can activate iPLA2γ and initiate antioxidant action affecting the whole cell. In other words, redox regulations originating either from the cytosol or mitochondria elevate the levels of H_2_O_2_ or other ROS, which directly activate mitochondrial iPLA2γ/UCP. Uncoupling diminishes the production of mitochondrial superoxide, and therefore, also the mitochondrial matrix manganese superoxide dismutase produces less H_2_O_2_. Consequently, the release of H_2_O_2_ and superoxide from mitochondria to the cytosol ceases, leaving spare cell antioxidant capacity that had previously been used to detoxify the mitochondrial portion of H_2_O_2_ and superoxide. The additional detoxification capacity is thus manifested in other cellular processes [18].

In previously published experiments, we provided challenges either blocking the iPLA2γ function or ablating UCP2 (studying tissues of the UCP2 knockout mice). We found that both challenges increase the acute oxidative stress in the lung and spleen, suggesting that the antioxidant synergy of UCP2 and iPLA2γ belongs to significant cytoprotective and anti-inflammatory mechanisms in these tissues. For example, the mechanism of iPLA2γ-dependent UCP2-mediated antioxidant protection was tested using lipopolysaccharide (LPS)-induced pro-inflammatory and pro-oxidative responses.The following acute influence on the overall oxidative stress was reflected by protein carbonylation in murine lung and spleen mitochondria and tissue homogenates [20]. Challenges included blocking the iPLA2γ function by the selective inhibitor R-BEL and removing UCP2 by genetic ablation. The results are consistent with the UCP2 plus iPLA2γ synergic antioxidant mechanisms in these tissues and support its contribution to cytoprotective mechanisms in vivo [20].

### 3.5. Mechanism of Suppression of Mitochondrial Superoxide Formation by the Action of ANT or Other SLC25 Family Proteins

In addition to uncoupling proteins, the mitochondrial ANT and other SLC25 mitochondrial carrier family members, namely aspartate/glutamate antiporter and the phosphate carrier, are involved in the uncoupling effect of FAs on mitochondria [12,52,76,77,78,79,80]. Specifically, the FA-stimulated respiration, sensitive to carboxyatractyloside, has been observed in mitochondria isolated from rodent skeletal muscle [76], liver [52,76,78,79], heart [81], kidney [80], and BAT [82]. A study by Bertholet et al. utilized a patch-clamp recording of ANT currents directly from inner mitochondrial membranes from various mouse tissues. It shows that H^+^ transport is an integral function of the mitochondrial ANT [12]. The ANT-mediated H^+^ current requires free FAs and resembles the H^+^ leak via the UCP1 found in BAT. The ADP/ATP exchange via ANT negatively regulates the H^+^ current but does not completely inhibit it. However, unlike for UCP1, FAs were suggested to bind within the ANT translocation pathway as cofactors rather than transported species [12]. However, more recent experimental findings from using purified recombinant ANT1 reconstituted in the planar lipid bilayers reveal that ANT1 mediates H^+^ transport only in the presence of long-chain FAs and the results support the FA cycling mechanism for H^+^ transport [83].

The ANT has also been suggested to participate in the suppression of mitochondrial superoxide formation. It has been shown that FAs are natural uncouplers preventing the generation of superoxide and H_2_O_2_ by isolated rat heart mitochondria oxidizing succinate, and carboxyatractyloside abolishes the effect of FAs [81].

### 3.6. Antioxidant Synergy of iPLA2γ and ANT or iPLA2γ and Other SLC25 Family Proteins

Because mitochondria in various tissues possess phospholipase A2 activity, it has been speculated that iPLA2-cleaved FAs may be the primary physiological source of FAs for ANT-dependent H^+^ currents [12]. Nevertheless, the direct evidence supporting the role of mitochondrial iPLA2 in ANT-mediated uncoupling and antioxidant function is scarce. Our results have shown that iPLA2-dependent increase in respiration and decrease in ΔΨ_m_ is largely sensitive to carboxyatractyloside and that ANT is mainly responsible for uncoupling initiated by redox-sensitive mitochondrial iPLA2 in isolated rat heart mitochondria [17].

The putative roles of iPLA2 activity and relevant FAs on ROS generation have also been studied by using rat brain mitochondria [84]. The mild uncoupling effect was simulated by low micromolar concentrations of docosahexaenoic acid (DHA), a major iPLA2 product in the brain. It has been shown that ANT partly mediates DHA-induced uncoupling, and low micromolar DHA concentrations diminish ROS generation under given conditions. However, this study also shows that racemic BEL, a pharmacological inhibitor of iPLA2s, can exert detrimental effects on energy-dependent functions of mitochondria [84].

Our studies, utilizing brain mitochondria isolated from wild-type (WT) and iPLA2γ-KO mice, are consistent with the antioxidant synergy of iPLA2γ and ANT in the brain. We found that low micromolar concentrations of TBHP induced an increase in respiration and a parallel decrease in mitochondrial superoxide and H_2_O_2_ production. These effects of TBHP were entirely prevented by bovine serum albumin and inhibited by a selective inhibitor of iPLA2γ and were absent in brain mitochondria isolated from iPLA2γ-KO mice. Moreover, the TBHP-induced effects were fully sensitive to carboxyatractyloside, indicating the primary participation of ANT [85].

Because the phospholipase iPLA2γ is a major mediator releasing oxidized aliphatic chains (hydroperoxyFAs) from cardiolipin [44] and the ANT participates in oleate hydroperoxide-induced uncoupling [80], we speculate that redox-sensitive iPLA2γ together with ANT may also serve antioxidant function initiated by mitochondrial lipid peroxidation (Figure 3 and Figure 4).

## 4. Redox Activation of iPLA2γ

### 4.1. Direct Activation of iPLA2γ by Oxidants

In terms of the molecular mechanism of activation of iPLA2γ by H_2_O_2_, both the direct redox and indirect redox mechanisms are possible. Our experiments that involve using recombinant iPLA2γ reconstituted in phospholipid vesicles show that hydroperoxides (both H_2_O_2_ and TBHP) can activate iPLA2γ directly, with a half-maximum activation constant AC_50_ corresponding to about 1.5 nmol H_2_O_2_ per nmol of the enzyme [19]. For cultured cells, we also confirmed the direct activation of iPLA2γ function by H_2_O_2_ [19].

Protein thiols are one of the few classes of biological molecules that H_2_O_2_ directly reacts with [86], and mitochondria are particularly rich in protein thiols, exceeding the concentrations of small molecular thiol pools, such as glutathione, by several-fold [87]. Redox regulation by thiol switching has been a long-standing candidate mechanism to support rapid adjustment of mitochondrial protein function at the posttranslational level [88]. Thus, accessible thiols (deprotonated to give rise to the reactive thiolate anion) allow reversible oxidation by H_2_O_2_ to sulfenic acid. Reactive sulfenic acids can be glutathionylated, form inter- or intramolecular disulfide bonds, or can be further oxidized to sulfinic or even sulfonic acids. Several additional thiol-redox modifications such as S-nitrosylation are possible [88,89,90,91]. The direct redox activation may also be propagated by proteins capable of SH relay, such as peroxiredoxins, directly affecting cysteine residues of the enzyme [6,7,92].

Moreover, lipid peroxidation and further decomposition of lipoperoxidation products often yield reactive electrophiles, including α,β-unsaturated aldehydes, malondialdehyde, hydroxyalkenals, oxoalkenals, epoxyalkenals, and γ-ketoaldehydes. The nucleophilic amino acids cysteine, histidine, and lysine are the most commonly modified by lipid-derived electrophiles [93,94].

According to our own computational predictions, human iPLA2γ contains Cys558 and Cys714, which are potentially vulnerable to oxidative modifications. Moreover, mouse iPLA2γ is also listed in the Oximouse database developed by Chouchani and colleagues [95], which defines and validates cysteine redox networks within each tissue (https://oximouse.hms.harvard.edu/, accessed on 19 February 2021). Cys553 and/or Cys708 of iPLA2γ were uncovered to be oxidized in several tissues of young and old mice, including the BAT, lung, spleen, and brain, confirming the redox modifications of iPLA2γ.

Alternatively, the mediated redox activation could be substantiated by the proteins capable of SH relay, such as peroxiredoxins, so that thiols of peroxiredoxin 3 (PRDX3) residing in the mitochondrial matrix are oxidized first, transferring this redox signal to oxidize thiols of iPLA2γ. In addition, other oxidant species could hypothetically interact with the iPLA2γ thiols and activate the enzyme. Finally, redox activation of the protein kinase-mediated signaling pathway, which activates iPLA2γ, may be plausible. The enzyme activation would be mediated through protein phosphorylation. Such a mechanism was indicated by the activation of iPLA2γ in experimental membranous nephropathy, where complement C5b-9-induces glomerular epithelial cell injury [96]. Thus, complement-mediated activation of iPLA2γ is mediated via ERK and p38 pathways, and phosphorylation of Ser-511 and/or Ser-515 plays a key role in the catalytic activity and signaling of iPLA2γ [96].

### 4.2. iPLA2γ vs. Lipid Hydroperoxides and Other Products of Lipid Peroxidation

Lipid peroxidation leads to structural alterations that impair membrane integrity [97]. The actual hydroperoxide group of lipid hydroperoxides moves to the lipid–water interface, as demonstrated by molecular dynamic simulation [98,99]. Early work on the mechanism of PLA2 catalytic activities studied lipid peroxidation and phospholipase A2 activity in liposomes composed of unsaturated phospholipids, and a direct correlation was found between the degree of lipid peroxidation and the extent of phospholipid hydrolysis [100]. The authors concluded that lipid peroxidation produces a general increase in membrane viscosity, which is associated with vesicle instability and enhanced PLA2 attack [100]. This can now be considered as an indirect mechanism of PLA2 activation because of lipid peroxidation or, alternatively, by the local structural alterations in a lipid bilayer. However, other experiments show that the increase in phospholipase A2 activity is mainly due to a preference for peroxidized phospholipid molecules as substrates and that peroxidation of host lipid does not significantly increase the rate of hydrolysis of nonoxidized lipids [101]. As Figure 4 depicts, hydroperoxide interaction with the thiols of iPLA2γ does not necessarily need to be mediated exclusively by “free H_2_O_2_”. It can also be mediated by the hydroperoxide groups of FAs esterified in membrane phospholipids. Our preliminary data have shown that lipid hydroperoxides can interact with iPLA2γ, resulting in similar enzyme activation [40]. It still requires to be determined whether iPLA2γ reduces lipid hydroperoxides upon its activation.

Moreover, activation of iPLA2γ by the local structural alterations of lipid bilayer could also hypothetically exist. This would happen if the lipid hydroperoxides were not reduced upon interaction with iPLA2γ. Although the structure of iPLA2γ is not known, one can speculate its mechanism of action is similar to iPLA2β. Within the iPLA2β dimer, both active sites provide an open space for phospholipids which easily reach the active centers. Speculatively, this might be further facilitated at the loci of lipid bilayer perturbations due to hydroperoxy FA and hydroxy FA, namely those of cardiolipins, since their polar groups are located at the lipid/water interface of phospholipid headgroups (Figure 3 and Figure 4C). As a result, an indirect redox mechanism could be mediated by the redox modification of a particular lipid component first, which would subsequently activate the iPLA2γ activity. If this mechanism exists, not only hydroperoxy FAs but also hydroxy FAs and any other acyl chains inducing the irregularities and structural alterations within the lipid bilayer are able to activate the enzyme.

In a complex cellular environment, H_2_O_2_ could initiate non-enzymatic lipid peroxidation via the Fenton reaction (which creates hydroxyl radical, capable of initiating lipid peroxidation) [102], and the resulting lipid hydroperoxides and subsequently derived reactive electrophiles might activate iPLA2γ. The active iPLA2γ may release the free hydroperoxy FAs (hydroxy FAs), which might activate its population further. The plausibility of such “self-activation” is based on our experiments with the recombinant iPLA2γ in proteoliposomes containing cardiolipin hydroperoxides [40]. Moreover, iPLA2γ was found to hydrolyze oxidized cardiolipin, releasing specifically 9-hydroxy-octadecenoic acid [44].

## 5. Dependence on Lipid Bilayer Structure and Cardiolipin

### Cardiolipin and iPLA2γ 

Cardiolipin, 1,3-bis(sn-3′-phosphatidyl)-sn-glycerol, represents a unique class of lipids, possessing typically one, but potentially two negative charges, within a small head group with typically four hydrocarbon tails. Two phosphatidic acid moieties connect with a glycerol backbone in the center, thus forming a dimeric structure. At pH around 7, only a single phosphate is ionized, which presumably allows cardiolipin to trap protons within its headgroup and thereby localize the proton pool near the surface of the inner membrane. The small headgroup having four hydrocarbon tails destabilizes the lipid bilayers by negative curvature stress [103].

The last steps in cardiolipin synthesis proceed in the matrix side of IMM, when cytidine diphosphate diacylglycerol (CDP-DAG), a product of CDP-DAG synthase (from phosphatidic acid and CTP as precursors), is converted to phosphatidylglycerol (PG). Subsequent PG and CDP-DAG condensation by the cardiolipin synthase produces cardiolipin [104]. Cardiolipin transacylation is subsequently mediated by the specific transacylase tafazzin (TAZ) located in the outer intermembrane space between OMM and IBM. Prior to this, the originally nascent synthesized cardiolipin situated in the inner, i.e., matrix, lipid leaflet of IBM must flip to the outer lipid leaflet. The earlier suggestion that iPLA2γ also participates in such cardiolipin remodeling by deacylation was questioned later [105].

Unlike other cellular membranes, mitochondrial membranes altogether contain ~22% of cardiolipin in addition to 37% of phosphatidylcholine (PChol), 31% of phosphatidylethanolamine (PE), and 6% of phosphatidylinositol (PI) [106]. IMM contains 20% of cardiolipin, which is even more concentrated in the crista junctions where OMM and IMM meet around the crista outlets, whereas yeast OMM contains only about 4% of cardiolipin [104]. The acyl chains are tissue-specific. In human heart cardiolipin, linoleoyl (C18:2) prevails, as well as cardiolipin with all four linoleoyl (C18:2) tails [107]. In the mouse brain, cardiolipins with (C18:1)_4_, (C18:1)_3_-(C20:4), and (C18:1)_3_-(C22:6) acyl chains dominated [108]. The mitochondrial isoform of nucleoside-diphosphate kinase D (NDPKD) resides in the outer intermembrane space, in the layer between OMM and IBM. Therein NDPKD transfers cardiolipin between IMM and OMM, providing the signal for mitophagy [103,109].

Cardiolipin is essential for mitochondrial cristae organization [110]. For example, the optical atrophy 1 (OPA1) protein ensures the fusion of both OMM and IMM and forms a “bottle cork” of cristae outlets. A short form of OPA1 (S-OPA1) cleaved from the long L-OPA1 by protease OMA1 interacts with cardiolipin, which is essential for correct cristae morphology and IMM fusion [111]. Similarly, Complex I [112], Complex IV [113], and ANT [114] require contacts with cardiolipin for their proper function, as well as the membrane F_O_ moiety of the ATP-synthase [115]. The crista outlets are surrounded by crista junctions where OMM and IMM mutually interact (Figure 2). These contacts are ensured by protein complexes termed MICOS join with the SAM complexes of OMM [116,117], as well as by the specific structure of cardiolipin.

Hydrolysis of tetraoleoyl(18:1)-cardiolipin by iPLA2β yielded the release of monolyso-cardiolipin and dilyso-cardiolipin [118]. There was no PLA1 activity towards dilyso-cardiolipin. In contrast, cardiolipin inhibited iPLA2 activity towards other phospholipids. Other experiments found specifically 9-hydroxy-octadecenoic acid upon the iPLA2γ-mediated hydrolysis of oxidized cardiolipin [44].

## 6. Fatty Acid–Mediated Signaling by iPLA2γ 

### 6.1. Mitochondrial FAs as Messengers in Information Signaling 

As pointed out above, FAs serve as the intramitochondrial messengers and initiators of mild uncoupling resulting in antioxidant action. Relationships between FA β-oxidation andthe concomitant enhanced superoxide formation, which is followed by the activation of iPLA2γ, have been manifested in several tissues, including skeletal muscle and heart. The best-documented example relates to theFA–stimulated insulin secretion (FASIS) in pancreatic β-cells. Here, besides a hypothetical direct redox-sensitive insulin secretion, amplification of the GPR40 pathway is provided by the intramitochondrial redox signaling. This is mediated by H_2_O_2_ resulting from ETFQOR-formed superoxide. H_2_O_2_ directly activates iPLA2γ, which subsequently cleaves fatty acids from phospholipids constituting mitochondrial membranes. The cleaved FAs migrate to the plasma membrane, where they additionally stimulate the GPR40 pathway of FASIS [19]. Note that such amplification accounts for ~60% of FASIS-generated insulin.

### 6.2. Mitochondrial Lysophospholipids as Messengers in Information Signaling 

Despite the detailed knowledge on phospholipid and cardiolipin synthesis and transfer of phospholipids between ER and mitochondrial membranes, there is no consensus, whether mitochondrial phospholipids are remodeled by mitochondrial acyltransferases such as glycerol-3-phosphate acyltransferase 1 and 2 (GPAT1,2) isoforms located on OMM and whether lysophospholipids are transported back to ER to be re-esterified [119].

We may speculate that such a redox-initiated cleavage of mitochondrial phospholipids by iPLA2γ may also simultaneously increase the H^+^ leak of the IMM. However, this has not been observed. The absolute requirement of the iPLA2γ-dependent H^+^ transport being mediated either by ANT1 or UCP2 suggests that the iPLA2γ catalytic activity is under strict regulation. Studies using purified recombinant iPLA2γ reconstituted in phospholipid vesicles are consistent with reversible direct activation of the enzyme by H_2_O_2_, suggesting a reversible oxidative modification of protein thiol(s).

### 6.3. Hydroperoxy FAs, Hydroxy FAs, and other Lipid Peroxidation Products as Messengers in Information Signaling 

The best-documented example of signaling by peroxidized forms of cardiolipin concerns the initiation of mitochondrial-dependent cell death, apoptosis. The consensus became that lipid peroxidation of cardiolipin and subsequent transfer of cardiolipin oxygenated forms to the OMM represents a signaling point of “no return” for initiation of apoptosis [109]. Perhaps OPA1 trimers in the crista outlets are dissociated in these early stages of apoptotic initiation [120] and allow two-dimensional diffusion of the intracristal membrane cardiolipin to IBM and the subsequent above described relocalization to OMM. All these changes result in the release of cytochrome c from mitochondria into the cytosol, initiating mitochondria-dependent apoptosis.

## 7. Physiological Cytoprotective and Regulatory Roles of iPLA2γ

### 7.1. Mitochondrial iPLA2γ in Heart Physiology and Pathology

Heart failure is associated with FA overload and oxidative stress from the accumulation of cardiotoxic lipid derivatives and oxidized fatty acid metabolites, including eicosanoids, docosanoids, and linoleic acid metabolites [35,121] together with a rapid increase in activity of certain mitochondrial iPLA2 [122]. Pharmacological inhibition of iPLA2 activity was found to attenuate mitochondrial phospholipid loss and prevent myocardial ischemia/reperfusion injury [123]. The specific role of iPLA2γ in the heart was elucidated by using cardiac myocyte-specific iPLA2γ knockout (CMiPLA2γKO) mice. Hearts of CMiPLA2γKO mice exhibit normal hemodynamic function, standard glycerophospholipid composition, and unchanged rates of mitochondrial respiration and ATP production but attenuated Ca^2+^-induced mitochondrial permeability transition pore (mPTP) opening [124].

In a subsequent study, Moon et al. demonstrated that heart failure activates specific myocardial mitochondrial phospholipases that increase Ca^2+^-dependent production of toxic hydroxyeicosatetraenoic acids (HETEs). The activity of phospholipases was attenuated so that a significant promotion was induced for the synthesis of protective epoxyeicosatrienoic acids (EETs) [29]. Mechanistically, HETEs activate the Ca^2+^-induced opening of the mPTP in failing myocardium, and the highly selective pharmacological blockade of either iPLA2γ or lipoxygenases attenuated mPTP opening in failing hearts. Surprisingly, the major mitochondrial phospholipase responsible for the Ca^2+^-activated release of arachidonic acid in mitochondria from non-failing hearts was identified as the cytosolic phospholipase A2ζ (cPLA2ζ, termed calcium-dependent phospholipase A2ζ in the respective manuscript). The cPLA2ζ is then responsible for protective EET production in healthy hearts and iPLA2γ producing HETEs in mitochondria from failing hearts [29].

In contrast to the identification of iPLA2γ as an essential mechanistic contributor of the mPTP and cardiac myocyte necrosis/apoptosis, the iPLA2γ may also serve essential physiological regulatory functions in the heart. In addition to the above-proposed participation of iPLA2γ in regulating cellular redox status, a mitochondrial iPLA2 has also been suggested to play a role in the propagation of cardioprotective signal [125]. Selective pharmacological blockage of mitochondrial iPLA2(s) interrupts redox-dependent activation of mitochondrial protein kinase Cε and subsequent opening of the mitochondrial ATP-sensitive K^+^ channel [125]. The data suggest that a spatiotemporal release of hydroperoxy FAs by mitochondrial iPLA2 is responsible for propagating redox signal during cardioprotection. However, more detailed in vivo studies are lacking, and the various potential roles of iPLA2γ in the heart have still not been fully elucidated.

### 7.2. Mitochondrial iPLA2γ in Brain Physiology and Pathology

In the central nervous system, phospholipases A2 have long been known to generate signals by lipid second messengers necessary for critical neuronal functions, including neurotransmitter release, long-term potentiation, and cognitive function [126]. Activation of PLA2s in cerebral ischemia has been correlated with increased lipid peroxidation [127,128], and iPLA2 was identified as the predominant phospholipase activity present in the rat hippocampus [126]. Subsequent studies utilizing iPLA2γ-KO mice identified the obligatory role of iPLA2γ in neuronal mitochondrial structure and function [129]. Moreover, iPLA2γ-KO mice are characterized by significant changes in hippocampal lipids, including cardiolipin content and molecular species composition, the presence of increased levels of oxidized lipid molecular species. In addition, the iPLA2γ-KO mice reveal markedly enlarged heteromorphic structures, which are particularly prominent in the hippocampus. The isolation of the enlarged structures identified them as degenerating mitochondria [129]. The results obtained from iPLA2γ-KO mice also emphasized the diverse array of biochemical and neuropathological abnormalities resulting from iPLA2γ loss of function, including disruption of iPLA2γ-mediated signaling and membrane lipid remodeling, mitochondrial degeneration, increased oxidative stress, and precipitation of neurologic dysfunction [129].

The role of iPLA2γ in the regulation of mitochondrial lipid peroxidation and mitochondrial dysfunction was studied by using a rotenone-induced rat model of Parkinson’s disease. Both in vivo and in vitro experiments revealed that iPLA2γ plays a critical part in keeping typical mitochondrial function. Decreasing iPLA2γ activity increases lipid oxidative stress accompanied by mitochondrial disorders [130]. Thus, neurodegeneration is strongly related to iPLA2γ activity, and when its activity is compromised, then the upregulation of the iPLA2γ activity can restore the mitochondrial membrane and function.

An additional role of iPLA2γ in the brain was shown by quantitative assessment of peroxidation and hydrolysis of mitochondrial cardiolipins, using global (phospho) lipidomics and redox lipidomics, to reveal and identify cardiolipin modifications during controlled cortical impact [131]. By comparing the in vitro and in vivo results with genetic manipulation of major cardiolipin metabolizing enzymes, iPLA2γ and tafazzin, it has been shown that the cardiolipin oxidation and cardiolipin hydrolysis act as mutually synergistically enhancing components of the pathogenic mechanism of mitochondrial damage in traumatic brain injury [131].

Moreover, recent ongoing progress on the antioxidant role of iPLA2γ in brain mitochondria isolated from wild-type and iPLA2γ-KO mice is consistent with an antioxidant role of iPLA2γ in the brain, utilizing a mechanism iPLA2γ-dependent, ANT-mediated attenuation of mitochondrial superoxide production by mild uncoupling [85].

### 7.3. Mitochondrial iPLA2γ in BAT Physiology and Pathology

Brown adipose tissue (BAT) and brown in white (brite) adipose tissue, also termed beige adipose tissue, are major sites of mammalian non-shivering thermogenesis, and mitochondrial UCP1, specific for these tissues, is the key factor for heat production (for review, see Reference [132]). Because UCP1 function is essentially dependent on free FAs in mitochondria, the discovery of N-terminal mitochondrial localization signal of iPLA2γ immediately suggested that iPLA2γ function could be connected to the function of UCPs and that iPLA2γ might contribute to integrate respiration and thermogenesis in BAT mitochondria [26]. In line with this hypothesis, the iPLA2γ-KO mice display significantly reduced cold tolerance, although the UCP1 mRNA expression slightly increased after cold induction, which may represent a compensatory mechanism to cope with altered heat generation [133].

Direct patch-clamp studies of the IMM of BAT mitochondria correlated the UCP1-mediated H^+^ currents with the activity of a certain mitochondrial PLA2 [13]. In line with the observation that FAs liberated from the IMM by PLA2 can activate UCP1, is the observation that iPLA2 inhibitor BEL inhibited isoproterenol-induced UCP1-dependent oxygen consumption [134]. 

The first detailed assessment of in vivo BAT metabolism of oxidants upon acute activation of thermogenesis is paralleled by elevated levels of mitochondrial superoxide, H_2_O_2_, and lipid hydroperoxides [135]. Moreover, the elevated production of mitochondrial oxidants is paralleled by a shift in a cysteine thiol redox status and leads to sulfenylation of UCP1 Cys253 [135]. Because of these findings, we have recently hypothesized that the elevated levels of oxidants stimulate the activity of redox-sensitive mitochondrial phospholipase iPLA2γ, which then provides nascent fatty acids for UCP1-mediated uncoupling, thus substantiating heat production [132]. Nevertheless, the specific roles for FAs and lysophospholipids resulting from the enzymatic activity of the mitochondrial phospholipase iPLA2γ in BAT mitochondria are yet to be elucidated.

## 8. Conclusions

Despite strenuous efforts by many laboratories to characterize the physiological and pathophysiological roles of phospholipases acting in or on mitochondria, our understanding of the participation of these phospholipases in the regulation of mitochondrial and cellular function remains limited. In the present review, we focused on clarifying the molecular mechanism of antioxidant synergy involving phospholipase iPLA2γ and selected fatty acid–translocating mitochondrial SLC25 family proteins.

The unique ability of iPLA2γ to cleave phospholipid acyl chains from both sn-1 and sn-2 positions allows for various ways of the spatiotemporal release of saturated and (poly)unsaturated fatty acids of diverse properties. The conditions under which the respective released fatty acids can act as regulators of mitochondrial H^+^ backflow into the mitochondrial matrix and subsequently as regulators of mitochondrial superoxide production were discussed. In addition, the phospholipid composition of the inner mitochondrial membrane, determined by the exclusive content of cardiolipin, allows iPLA2γ to release specific lipid species, which may subsequently act as unique mitochondria-derived lipid mediators of cellular signaling.

A redox-dependent regulation of iPLA2γ has been identified as a significant key feature required for the function and antioxidant action of iPLA2γ. Although some progress has been made in clarifying the molecular mechanism of redox activation of iPLA2γ, there are still many questions that remain unanswered. These include the identification of the molecular species responsible for the activation of iPLA2γ and identification of the respective amino acid residues of iPLA2γ, which undergo oxidative posttranslational modification. The detailed knowledge of the molecular mechanism(s) responsible for the regulation of iPLA2γ will help us understand and predict the still unexplored cytoprotective and regulatory roles of mitochondrial iPLA2γ in the context of cellular physiology and pathology.

## Figures and Tables

**Table 1:** Overview of selected intracellular calcium-independent phospholipases A2 (patatin-like phospholipase domain-containing proteins, PNPLA), calcium-dependent cytosolic phospholipases A2 (cPLA2s), and low-molecular-weight secreted phospholipases A2 (sPLA2s).

Phospholipase A2			Localization	Acts on Membrane	Targets	Cleavage Products	Main Functions
Group	Gene Name	Protein					
VIE	PNPLA2	iPLA2ζ/ATGL	Adipocyte: Cytosolic	Plasma membrane OMM outer leaflet	Neutral lipid lipase: triacylglycerols	FAs + diacylglycerols	Nutrition regulation, Hydrolysis of triglycerides
VID	PNPLA3	iPLA2ε	Hepatocyte: Cytosolic	Plasma membrane OMM outer leaflet	Neutral lipid lipase: triacylglycerols	FAs + diacylglycerols	Phosphatidic acid generation, Acyl-chain remodeling of triglycerides
VIF	PNPLA4	iPLA2η	Ubiquitous: Cytosolic	Plasma membrane OMM outer leaflet	sn-1, sn-2 PL, LysoPL, triacylglycerols	FAs + LysoPL, FAs + diacylglycerols	Triacylglycerol lipase activity, Cytoprotection
VIC	PNPLA6, PNPLA7	iPLA2δ, iPLA2θ	Neurons: Cytosolic	Plasma membrane OMM outer leaflet	ER, Golgi sn-1, sn-2 PL, LysoPL	FAs + LysoPL	Neuroprotection, Intracellular membrane trafficking
VIB	PNPLA8	iPLA2γ	Ubiquitous: Mitochondrial Peroxisomal	IMM (outer or inner leaflet) OMM inner leaflet Peroxisome	sn-1, sn-2 PL, cardiolipin, hydroperoxy-PL, hydroperoxy-CL	Saturated or unsaturated FAs, PUFAs (or hydroperoxy) + PUFAlysPL or LysoPL	Antioxidant, Lipid second messengers, Eicosanoid signaling, Cardiolipin remodeling
VIA	PNPLA9	iPLA2β	Ubiquitous: Mitochondrial Cytosolic	OMM (outer or inner leaflet) IMM (outer or inner leaflet)	sn-1, sn-2 PL, cardiolipin, hydroperoxy-PL, hydroperoxy-CL	Saturated or unsaturated FAs, PUFAs (or hydroperoxy) + PUFAlysPL or LysoPL	Cellular membrane homeostasis, Mitochondrial integrity, Signal transduction
IV	PLA2G4	cPLA2s	Cytosolic	Plasma membrane inner leaflet; OMM outer leaflet	sn-2 PL	FAs, PUFAs	Membrane lipid remodeling, Biosynthesis of lipid mediators, inflammatory response
IVF	PLA2G4F	cPLA2ζ	Cytosolic, Mitochondrial	OMM (outer or inner leaflet) IMM (outer or inner leaflet)	sn-2 PL	FAs, PUFAs	Biosynthesis of lipid mediators, Arachidonate release, Cardioprotective eicosanoids
I, II, III V, X, XII	PLA2G1, PLA2G2, PLA2G3, PLA2G5, PLA2G10, PLA2G12	sPLA2	Extracellular matrix	Plasma membrane outer leaflet	sn-2 PL	FAs, PUFAs	Extracellular matrix remodeling, Lipid mediator secretion, Digestion, Immunity
